# Association of e-Cigarette Use With Discontinuation of Cigarette Smoking Among Adult Smokers Who Were Initially Never Planning to Quit

**DOI:** 10.1001/jamanetworkopen.2021.40880

**Published:** 2021-12-28

**Authors:** Karin A. Kasza, Kathryn C. Edwards, Heather L. Kimmel, Andrew Anesetti-Rothermel, K. Michael Cummings, Raymond S. Niaura, Akshika Sharma, Erin M. Ellis, Rebecca Jackson, Carlos Blanco, Marushka L. Silveira, Dorothy K. Hatsukami, Andrew Hyland

**Affiliations:** 1 Department of Health Behavior, Roswell Park Comprehensive Cancer Center, Buffalo, New York; 2 Westat Inc, Rockville, Maryland; 3 National Institute on Drug Abuse, National Institutes of Health, Bethesda, Maryland; 4 Center for Tobacco Products Food and Drug Administration, Silver Spring, Maryland; 5 Medical University of South Carolina, Charleston; 6 New York University, New York; 7 Kelly Government Solutions, Rockville, Maryland; 8 Masonic Cancer Center, University of Minnesota, Minneapolis

## Abstract

**Importance:**

Cigarette smokers not planning to quit are often overlooked in population studies evaluating the risk-benefit potential of electronic nicotine delivery products (e-cigarettes).

**Objective:**

To evaluate whether e-cigarette use is associated with discontinuing cigarette smoking among smokers who were initially never planning to quit.

**Design, Setting, and Participants:**

This cohort study used US nationally representative data from the longitudinal Population Assessment of Tobacco and Health Study (waves 2-5 conducted between October 2014 and November 2019), with participants evaluated in 3 pairs of interviews. Adult daily cigarette smokers initially not using e-cigarettes and with no plans to ever quit smoking for good (2489 observations from 1600 individuals) were included.

**Exposures:**

e-Cigarette use (ie, daily use, nondaily use, or no use) at follow-up interview among smokers not using e-cigarettes at baseline interview.

**Main Outcomes and Measures:**

The main outcomes were discontinuation of cigarette smoking (ie, no cigarette smoking) and discontinuation of daily cigarette smoking (ie, no daily cigarette smoking) at follow-up interview. Generalized estimating equations were used to evaluate the association between the exposure and each outcome, controlling for demographic characteristics and cigarettes smoked per day at baseline interview; all estimates were weighted.

**Results:**

The weighted population of adult daily cigarette smokers who were not using e-cigarettes and had no plans to ever quit smoking, based on data from 1600 participants, was 56.1% male (95% CI, 53.4%-58.7%), 10.1% Hispanic (95% CI, 8.2%-12.3%), 10.1% non-Hispanic Black (95% CI, 8.7%-11.7%), 75.6% non-Hispanic White (95% CI, 72.9%-78.2%), and 4.2% of other non-Hispanic race (95% CI, 3.3%-5.4%); 29.3% were aged 55 to 69 years (95% CI, 26.2%-32.6%), 8.9% were aged 70 years or older (95% CI, 6.8%-11.5%), 36.8% did not graduate from high school (95% CI, 34.1%-39.6%), 55.2% had an annual household income of less than $25 000 (95% CI, 52.3%-58.1%), 37.6% smoked 20 to 29 cigarettes per day (95% CI, 34.7%-40.6%), and 12.7% smoked 30 or more cigarettes per day (95% CI, 10.9%-14.7%). Overall, 6.2% of the population (95% CI, 5.0%-7.5%) discontinued cigarette smoking. Discontinuation rates were higher among those who used e-cigarettes daily (28.0%; 95% CI, 15.2%-45.9%) compared with not at all (5.8%; 95% CI, 4.7%-7.2%; adjusted odds ratio [aOR], 8.11; 95% CI, 3.14-20.97). Furthermore, 10.7% (95% CI, 9.1%-12.5%) discontinued daily cigarette smoking, with higher rates of discontinuation observed among those who used e-cigarettes daily (45.5%; 95% CI, 27.4%-64.9%) compared with not at all (9.9%; 95% CI, 8.2%-11.8%; aOR, 9.67; 95% CI, 4.02-23.25). Nondaily e-cigarette use was not associated with cigarette discontinuation (aOR, 0.53; 95% CI, 0.08-3.35) or daily cigarette discontinuation (aOR, 0.96; 95% CI, 0.44-2.09).

**Conclusions and Relevance:**

In this cohort study, daily e-cigarette use was associated with greater odds of cigarette discontinuation among smokers who initially had no plans to ever quit smoking. These findings support the consideration of smokers who are not planning to quit when evaluating the risk-benefit potential of e-cigarettes for smoking cessation in the population.

## Key Points

**Question:**

Is e-cigarette use associated with discontinuation of cigarette use among smokers initially not planning to ever quit?

**Findings:**

In this US nationally representative cohort study of 1600 adult daily cigarette smokers who did not initially use e-cigarettes and had no plans to ever quit smoking, subsequent daily e-cigarette use was significantly associated with an 8-fold greater odds of cigarette discontinuation compared with no e-cigarette use.

**Meaning:**

These findings call for consideration of smokers who are not planning to quit when evaluating the risk-benefit potential of e-cigarettes for smoking cessation in the population.

## Teaser

This cohort study evaluates whether e-cigarette use is associated with the discontinuation of cigarette smoking among US adult smokers who were initially never planning to quit.

## Introduction

Most published studies that investigate whether use of electronic nicotine delivery products (e-cigarettes) can help cigarette smokers quit smoking have been restricted to smokers who are planning to quit, or they have not considered smokers’ quit intentions.^1^ However, a recent study using data from the US nationally representative Population Assessment of Tobacco and Health (PATH) Study looked at daily cigarette smokers who were not planning to ever quit smoking and found that subsequent daily use of e-cigarettes was positively associated with change in intentions to quit cigarette use.^2^ A long-standing theory suggests that taking even a first step toward contemplating quitting smoking can have a positive impact on net cigarette cessation rates^3^; thus, evaluation of factors associated with cigarette discontinuation among smokers not planning to quit is important to understanding the range of potential impacts of e-cigarette use on net cigarette cessation. In this study, we assessed whether use of e-cigarettes was associated with cigarette discontinuation among adult daily cigarette smokers who were initially not planning to ever quit smoking and were initially not using e-cigarettes. We used 4 waves of PATH Study data collected in 2014-2015, 2015-2016, 2016-2017, and 2018-2019 to extend the population-based e-cigarette use and cigarette discontinuation literature to include this segment of the smoking population.

## Methods

### Participants

The PATH Study is an ongoing, nationally representative, longitudinal cohort study of youth and adults in the US that collects self-reported information on tobacco-use behaviors, attitudes and beliefs, and health outcomes. Data were collected using audio computer-assisted self-interviews administered in English or Spanish between October 2014 and October 2015 (wave 2), October 2015 and October 2016 (wave 3), December 2016 and January 2018 (wave 4), and 2 years later between December 2018 and November 2019 (wave 5). Data from wave 1 were not included owing to key changes in relevant items, although weighted analyses in the present study represent those in the population at the time of wave 1 (see Statistical Analysis section). The PATH Study was conducted by Westat and approved by the Westat Institutional Review Board. All adult respondents aged 18 years or older provided written informed consent. For the adult interview, the overall weighted response rate at wave 1 was 74.0%, at wave 2 was 83.2%, at wave 3 was 78.4%, at wave 4 was 73.5%, and at wave 5 was 69.4%.^4^ Further details regarding the PATH Study design and methods^5,6,7^ and overall demographic and tobacco use distributions^8^ are published elsewhere. Details on interviewing procedures, questionnaires, sampling, weighting, response rates, and accessing the data are also published elsewhere.^4^ This report followed the Strengthening the Reporting of Observational Studies in Epidemiology (STROBE) reporting guideline for cohort studies.

We conducted analyses among the 17.0% (95% CI, 16.0%-18.0%) of adult (aged 18 years or older) cigarette smokers in the US who were daily cigarette smokers, not using e-cigarettes (though they may have been using other tobacco products), had no plans to ever quit smoking for good, and had follow-up data on e-cigarette use status and cigarette discontinuation (2489 observations contributed by 1600 individuals). A total of 908 individuals were lost to follow-up over the course of the study period. Those with missing data on educational attainment or annual household income were included in analyses as valid unknown groups; those with missing data on other covariates were excluded from this study using listwise deletion (35 individuals). To adjust for complex study design characteristics (eg, oversampling) and attrition, all estimates were weighted and represent the resident population of the US aged 18 years or older at the time of data collection who were in the civilian, noninstitutionalized population in 2013-2014.^8^

### Measures

#### Sample-Defining Measures and Key Variable Measure

At each interview, respondents were asked separately whether they currently smoke cigarettes, whether they currently use e-cigarettes (ie, any electronic nicotine product), and whether they plan to ever quit cigarettes or tobacco for good. Exact item wordings are provided in Table 1. We restricted our analysis to those who at baseline assessment were currently smoking cigarettes every day, were currently using e-cigarettes not at all, and currently did not plan to ever quit cigarettes or tobacco for good. Our key variable measure was e-cigarette use at the follow-up wave using a 3-level e-cigarette use variable at follow-up: (1) no use, (2) nondaily use, or (3) daily use (Table 1).

**Table 1.** Measures

Measures	Categorizations	Questions and responses used in categorizations
Sample-defining measures (assessed at baseline wave of each wave pair)		
Cigarette smoking status	Daily smoker: smokes cigarettes every day	“Do you now smoke cigarettes…” with response options: every day/some days/not at all
Quit intentions	No intentions: does not plan to ever quit cigarettes/tobacco for good^a^	“Do you plan to ever quit [cigarettes/tobacco] for good?” with responses options: yes/no
e-Cigarette use status	Nonuser: does not use e-cigarettes at all	“Do you now use [e-cigarettes]…” with response options: every day/some days/not at all^b^
Predictor measure (assessed at follow-up wave among those who were e-cigarette nonusers at baseline wave)		
e-Cigarette use status	(1) No e-cigarette use (2) Nondaily e-cigarette use (3) Daily e-cigarette use	“Do you now use [e-cigarettes]…” with response options: every day/some days/not at all^b^
Outcome measures (assessed at follow-up wave among those who were daily smokers and had no intentions to quit at baseline wave)		
Cigarette discontinuation at follow-up	(1) Cigarette discontinuation: did not smoke in the past 12 mo or is currently smoking not at all (2) Cigarette smoker: is currently smoking every day or some days	“In the past 12 months, have you smoked a cigarette, even 1 or 2 puffs?” with response options: yes/no “Do you now smoke cigarettes…” with response options: every day/some days/not at all
Discontinuing daily cigarette smoking at follow-up	(1) Discontinuing daily cigarette smoking: did not smoke in the past 12 mo, or is currently smoking not at all, or is currently smoking some days (2) Daily cigarette smoker: is currently smoking every day	“In the past 12 months, have you smoked a cigarette, even 1 or 2 puffs?” with response options: yes/no “Do you now smoke cigarettes…” with response options: every day/some days/not at all

^a^ Cigarette smokers who were current users of any other non–e-cigarette tobacco product and had ever used that tobacco product fairly regularly were asked about intending to quit using tobacco rather than specifically about intending to quit smoking cigarettes.

^b^ e-Cigarettes refers to all e-products (ie, e-cigarettes, e-cigars, e-pipes, and e-hookah).

#### Outcome Measures

We defined cigarette discontinuation at follow-up assessment as having not smoked cigarettes in the past 12 months or currently smoking not at all. We defined discontinuing daily cigarette smoking at follow-up assessment as having not smoked in the past 12 months, currently smoking not at all, or currently smoking some days. Exact item wordings and variable categories are provided in Table 1.

#### Covariates

We assessed biological sex, race and ethnicity (Hispanic, non-Hispanic Black, non-Hispanic White, and multiracial groups or other non-Hispanic racial groups, including American Indian or Alaska Native, Asian Indian, Chinese, Filipino, Japanese, Korean, Vietnamese, other Asian, Native Hawaiian, Guamanian or Chamorro, Samoan, and other Pacific Islander), age group (18-24, 25-39, 40-54, 55-69, and ≥70 years), educational attainment (less than high school/general equivalency diploma, high school graduate, some college/associate’s degree, bachelor's degree or more, or unknown), annual household income (<$25 000, $25 000-$74 999, ≥$75 000, or unknown), and cigarettes smoked per day (<10, 10-19, 20-29, or ≥30 cigarettes per day).

### Statistical Analysis

We used generalized estimating equations to evaluate the association between time-varying e-cigarette use and time-varying cigarette discontinuation and discontinuation of daily cigarette smoking (so as to assess both the transition away from smoking altogether and the transition away from daily smoking in particular) using 3 wave pairs: waves 2-3, waves 3-4, and waves 4-5. Generalized estimating equations allow for the assessment of change between baseline and follow-up from all wave pairs in a single analysis while statistically controlling for interdependence among observations contributed by the same individuals.^9,10^ A parallel set of sensitivity analyses were also conducted in which we excluded 4 individuals who participated in waves 2-3, quit in wave 3, and participated again in waves 4-5. We used generalized estimating equation logistic regression models specifying unstructured covariance and within-person correlation matrices and a binomial distribution of the dependent variable using the logit link function. Analyses were adjusted for demographic characteristics (all were time-varying except for biological sex and race and ethnicity, which were time-invariant), cigarettes smoked per day (time-varying), and wave pair (as a time-varying categorical variable). The inclusion of wave pair in the analyses enabled us to control for time effects in the association between e-cigarette use and cigarette discontinuation. Analyses were weighted using the PATH Study W5 all-waves weights to produce nationally representative estimates. Variances were computed using the balanced repeated replication method^11^ with Fay adjustment set to 0.3.^12^ We set an a priori level of significance of *P <* .05, and hypothesis tests were 2-sided. Analyses were conducted using Stata, version 16.0 (StataCorp LP), using the svy suite of commands and adapting SAS macro code to run weighted generalized estimating equation analyses and calculate adjusted odd ratios (aORs) and 95% CIs.^13^ Analyses were run on the W2–W5 Restricted Use Files.^4^

## Results

The composition of the US population of adult daily cigarette smokers, based on data from 1600 adult participants, who were not using e-cigarettes and had no plans to ever quit smoking for good was 56.1% male (95% CI, 53.4%-58.7%); 10.1% Hispanic (95% CI, 8.2%-12.3%), 10.1% non-Hispanic Black (95% CI, 8.7%-11.7%), 75.6% non-Hispanic White (95% CI, 72.9%-78.2%), and 4.2% of other non-Hispanic race (95% CI, 3.3%-5.4%); 7.2% (95% CI, 6.3%-8.2%) were aged 18 to 24 years, 24.3% (95% CI, 21.8%-26.9%) were aged 25 to 39 years, 30.4% (95% CI, 27.0%-34.0%) were aged 40 to 54 years, 29.3% (95% CI, 26.2%-32.6%) were aged 55 to 69 years, 8.9% (95% CI, 6.8%-11.5%) were aged 70 years or older; 36.8% did not graduate from high school (95% CI, 34.1%-39.6%), 33.7% graduated from high school without further education (95% CI, 30.6%-37.0%), 22.9% completed some college or an associate degree without further education (95% CI, 20.1%-25.9%), 6.0% completed a bachelor degree or more education (95% CI, 4.5%-7.9%), and 0.7% had unknown educational attainment (95% CI, 0.4%-1.3%); 55.2% had an annual household income of less than $25 000 (95% CI, 52.3%-58.1%), 30.4% had an annual income of $25 000-$74 999 (95% CI, 27.8%-33.1%), 6.7% had an annual income of $75 000 or more (95% CI, 5.4%-8.4%), and 7.7% had an unknown annual income (95% CI, 6.2%-9.4%); 20.5% smoked fewer than 10 cigarettes per day (95% CI, 18.3%-23.0%), 29.2% smoked 10 to 19 cigarettes per day (95% CI, 26.3%-32.2%), 37.6% smoked 20 to 29 cigarettes per day (95% CI, 34.7%-40.6%), and 12.7% smoked at least 30 cigarettes per day (95% CI, 10.9%-14.7%). Of note, the estimate of 0.7% for individuals who had unknown educational attainment should be interpreted with caution because it has low statistical precision. It is based on a denominator sample size less than 50, or the coefficient of variation of the estimate or its complement is larger than 30%.

In the study population overall, 6.2% of adult daily cigarette smokers who were not using e-cigarettes and had no plans to ever quit smoking for good were not smoking cigarettes at all at follow-up (95% CI, 5.0%-7.5%). The odds of cigarette discontinuation were significantly higher among those who used e-cigarettes daily (28.0%; 95% CI, 15.2%-45.9%) compared with those who did not use e-cigarettes at all (5.8%; 95% CI, 4.6-7.2; aOR, 8.11; 95% CI, 3.14-20.97), while the odds of cigarette discontinuation among those who used e-cigarettes nondaily did not statistically differ from those who did not use e-cigarettes at all (Table 2). Overall, 10.7% of adult daily cigarette smokers who were not using e-cigarettes and had no plans to ever quit smoking for good discontinued daily cigarette smoking (95% CI, 9.1%-12.5%). The odds of discontinuing daily cigarette smoking were higher among those who used e-cigarettes daily (45.5%; 95% CI, 27.4%-64.9%) compared with those who did not use e-cigarettes at all (9.9%; 95% CI, 8.2%-11.8%; aOR, 9.67, 95% CI, 4.02-23.25), while the odds of discontinuing daily cigarette smoking among those who used e-cigarettes nondaily (10.2%; 95% CI, 5.8%-17.3%) did not statistically differ from those who did not use e-cigarettes at all (aOR, 0.96; 95% CI, 0.44-2.09) (Table 2).

**Table 2.** Cigarette Discontinuation and Discontinuing Daily Cigarette Smoking at Follow-up Wave, Among Daily Cigarette Smokers Who Had No Plans to Ever Quit for Good and Who Were Not Using e-Cigarettes at Baseline Wave, as a Function of e-Cigarette Use at Follow-up Wave^a^

e-Cigarette use at follow-up	Cigarette discontinuation at follow-up wave (ie, no cigarette smoking)		Discontinuing daily cigarette smoking at follow-up wave (ie, no daily cigarette smoking)	
	No. of observations (%) [95% CI]	aOR (95% CI)^b^	No.of observations (%) [95% CI]	aOR (95% CI)^b^
Overall (n = 2489)	158 (6.2) [5.0-7.5]	NA	271 (10.7) [9.1-12.5]	NA
No e-cigarette use (n = 2273)	138 (5.8) [4.7-7.2]	1 [Reference]	228 (9.9) [8.2-11.8]	1 [Reference]
Nondaily e-cigarette use (n = 156)	3 (3.1) [0.8-11.1]^c^	0.53 (0.08-3.35)	16 (10.2) [5.8-17.3]	0.96 (0.44-2.09)
Daily e-cigarette use (n = 60)	17 (28.0) [15.2-45.9]	8.11 (3.14-20.97)	27 (45.5) [27.4-64.9]	9.67 (4.02-23.25)

Abbreviations: aOR, adjusted odd ratios; NA, not applicable.

^a^ Numbers are unweighted and reflect numbers of observations; percentages, aORs, and 95% CIs are weighted using the PATH wave 5 all-waves weights for longitudinal analyses. Sample includes those who aged into the adult cohort over the course of the study period.

^b^ Analyses were adjusted for biological sex, race and ethnicity, age group, educational attainment, annual household income, cigarettes smoked per day, and wave pair; all covariates were assessed at baseline wave of each wave pair; generalized estimating equation models were fitted specifying the unstructured covariance and within-person correlation matrices, Wald χ^2^_22_ of 95.4 (*P* < .001) for the cigarette discontinuation model; Wald χ^2^_22_ of 170.6 (*P* < .001) for the daily cigarette discontinuation model.

^c^ Estimate should be interpreted with caution because it has low statistical precision. It is based on a denominator sample size of less than 50, or the coefficient of variation of the estimate or its complement is larger than 30%.

For the parallel set of sensitivity analyses in which we excluded 4 individuals who participated in W2-W3, quit in W3, and participated again in W4-W5, findings for the cigarette discontinuation outcome were an aOR of 8.65 (95% CI, 3.39-22.10) for daily e-cigarette use compared with no e-cigarette use and aOR of 0.58 (95% CI, 0.09-3.68) for nondaily e-cigarette use compared with no e-cigarette use. Findings for the daily cigarette discontinuation outcome were an aOR of 9.99 (95% CI, 4.14-24.09) for daily e-cigarette use compared with no e-cigarette use and an aOR of 1.00 (95% CI, 0.46-2.16) for nondaily e-cigarette use compared with no e-cigarette use.

## Discussion

This study focused on a group of daily smokers who were not initially planning to ever quit smoking and who did not use e-cigarettes; results showed that those who subsequently used e-cigarettes every day experienced an 8-fold higher odds of cigarette discontinuation compared with those who did not use e-cigarettes at all. Smokers with no plans to ever quit smoking tend to smoke more cigarettes per day and have lower educational attainment and household income compared with their counterparts who do plan to quit^2,14^ and are also often overlooked in the population-based e-cigarette use and cigarette discontinuation literature.^1^ Our findings here suggest that such smokers should be specifically considered when evaluating the risk-benefit potential of e-cigarettes for smoking cessation in the population.

Prior population-based work has shown that intentions to quit cigarettes change alongside uptake of e-cigarette use,^2^ suggesting a possible mechanism underlying our findings. Specifically, the association between e-cigarette use and change in quit intentions could expand the pool of smokers engaged in cessation efforts and ultimately increase overall cessation rates. Indeed, an experimental study among smokers not planning to quit found use of e-cigarettes to be associated with increased plans to quit,^15^ and an ecological momentary assessment found that giving e-cigarettes to smokers with no quit intentions was associated with reductions in cigarette smoking when e-cigarettes were used frequently.^16^ Further, our findings are consistent with clinical trials showing that giving nicotine replacement therapy (another type of nicotine delivery product) to smokers not planning to quit is associated with increases in overall smoking cessation rates.^17,18,19^ It is also possible, however, that change in quit intentions may not explain the association found between uptake of e-cigarette use and cigarette discontinuation; research from Hughes et al^20^ on the natural history of smokers’ efforts to quit smoking shows that intentions to quit smoking can change quickly and repeatedly and may not be a good indicator of behavior.

### Limitations

Limitations of our study include the lack of ability to evaluate whether quit intentions changed after uptake of e-cigarettes, and we did not assess whether any changes in quit intentions mediated the association between e-cigarette uptake and cigarette discontinuation; thus, future work is important to understand the causal mechanisms underlying our findings. Further, the collection of qualitative data can be useful to informing possible processes that may contribute to discontinuing smoking among this group of smokers. We also note that there may be self-selection differences between those who subsequently used e-cigarettes and those who did not, which were not assessed here. Another limitation is that we had relatively small sample sizes, although weighting ensured the sample was representative of the US population. We also did not assess long-term patterns of subsequent relapse and requitting. The e-cigarette marketplace has also changed since our study period of 2014 through 2019.

## Conclusions

Although controlled experimental studies have shown associations between e-cigarette use and smoking cessation among smokers without plans to quit, it is important to identify whether similar associations exist in a real-world context. This cohort study found an association between daily e-cigarette use and cigarette discontinuation among daily smokers in the US population who initially had no plans to ever quit smoking in their lifetimes. These findings call for consideration of smokers who are not planning to quit when evaluating the risk-benefit potential of e-cigarettes for smoking cessation in the population. Further, given the growing popularity of e-cigarettes in the US, and considering smokers’ very low interest in using traditional smoking cessation medications, future research into the comparative reach and effectiveness of different types of products will be important to informing clinical and regulatory strategies to increase cigarette discontinuation rates at the population level.
